# Dendritic Cells Pulsed with Leukemia Cell-Derived Exosomes More Efficiently Induce Antileukemic Immunities

**DOI:** 10.1371/journal.pone.0091463

**Published:** 2014-03-12

**Authors:** Ye Yao, Chun Wang, Wei Wei, Chang Shen, Xiaohui Deng, Linjun Chen, Liyuan Ma, Siguo Hao

**Affiliations:** 1 Department of Hematology, Xinhua Hospital Affiliated to Shanghai Jiaotong University School of Medicine, Shanghai, China; 2 Department of Hematology, The First People's Hospital of Shanghai Affiliated to Shanghai Jiaotong University, Shanghai, China; Technische Universitaet Muenchen, Germany

## Abstract

Dendritic cells (DCs) and tumor cell-derived exosomes have been used to develop antitumor vaccines. However, the biological properties and antileukemic effects of leukemia cell-derived exosomes (LEXs) are not well described. In this study, the biological properties and induction of antileukemic immunity of LEXs were investigated using transmission electron microscopy, western blot analysis, cytotoxicity assays, and animal studies. Similar to other tumor cells, leukemia cells release exosomes. Exosomes derived from K562 leukemia cells (LEX_K562_) are membrane-bound vesicles with diameters of approximately 50–100 μm and harbor adhesion molecules (*e.g.*, intercellular adhesion molecule-1) and immunologically associated molecules (*e.g.*, heat shock protein 70). In cytotoxicity assays and animal studies, LEXs-pulsed DCs induced an antileukemic cytotoxic T-lymphocyte immune response and antileukemic immunity more effectively than did LEXs and non-pulsed DCs (*P*<0.05). Therefore, LEXs may harbor antigens and immunological molecules associated with leukemia cells. As such, LEX-based vaccines may be a promising strategy for prolonging disease-free survival in patients with leukemia after chemotherapy or hematopoietic stem cell transplantation.

## Introduction

In patients with leukemia, the presence of minimal residual leukemia cells (MRLs) after chemotherapy and hematopoietic stem cell transplantation (HSCT) is a major cause of disease recurrence [1]. At present, eradication of MRLs is achieved with high-dose chemotherapy and allogeneic HSCT (allo-HSCT). The graft-versus-leukemia effects of allogeneic lymphocytes contribute to the antileukemic effect of allo-HSCT. However, these antileukemic immunological effects are nonspecific; as such, they are inefficient and can cause serious graft-versus-host disease, which contributes to morbidity after allo-HSCT. Several clinical and pre-clinical studies have identified immunotherapy as an approach to eliminate MRLs after chemotherapy and transplantation in order to reduce and prevent leukemia relapse. Indeed, several clinical studies have demonstrated the effectiveness of leukemia immunotherapy [2]–[4]. However, immunotherapy is limited by the lack of reliable leukemia-associated antigens. Therefore, it is important to identify leukemia cell-associated antigens in order to develop immunotherapies for leukemia and other hematologic malignancies.

Exosomes (EXOs) are vesicles that harbor multiple cell-membrane molecules and other proteins secreted by eukaryotes [5], [6]. EXOs are secreted by several cell types, particularly hematopoietic cells, including antigen-presenting cells such as dendritic cells (DCs), lymphocytes, mast cells, enterocytes, and tumor cells [2].

Recently, tumor cell-derived EXOs (TEXs) have attracted attention as a source of tumor antigens for use in vaccines [7]–[9]. TEXs harbor tumor-related antigens and can induce potent antitumor immune responses [5], [10]. TEXs isolated from malignant effusions can transfer tumor antigens to DCs to induce specific cytotoxic T-lymphocyte (CTL) responses and antitumor immunity [11]–[14]. Therefore, TEXs may be a source of tumor antigens for antitumor immunotherapy. However, a previous study reported that TEXs can induce antigen-specific tolerance through T-cell apoptosis and suppression of T-cell receptor/CD3-zeta by Fas ligand-containing EXOs from ovarian tumors [15]. In contrast, ours and other studies have demonstrated that EXOs secreted by tumor peptide-pulsed DCs can induce a specific antitumor response. Specifically, DCs can take up TEXs and the antigens harbored within, thus inducing strong antitumor immunity [16], [17]. These studies have demonstrated that TEXs and DCs can be implemented as cancer immunotherapy [4], [5]. The K562 cell line is a human chronic myelogenous leukemia cell line that contains the *BCR*:*ABL* fusion gene [18]. In this study, we investigated the biological characteristics of EXOs derived from K562 leukemia cells (LEX_K562_) and their ability to induce antileukemic immunity. Our results revealed that LEX_K562_ harbor the BCR-ABL fusion protein, which is expressed in the original K562 cell line. Furthermore, our results suggested that LEX can be taken up by DCs *in vitro* and that LEX-pulsed DCs induce a stronger antigen-specific antileukemic CTL immune response *in vivo*.

## Materials and Methods

### Reagents, cell lines, and animals

The research on “Dendritic cells pulsed with leukemia cell-derived exosomes induce more efficiently anti-leukemia immunities” will be carried out by Prof. Siguo Hao's research team, this research mainly focus on the biological properties and its anti-leukemia immunities of Dendritic cells pulsed with leukemia cell-derived exosomes. In this study, DBA/2 will be used as animal model and immunized with Dendritic cells pulsed with leukemia cell-derived exosomes and then mouse will be challenged with L1210 leukemia cells and the incidence of tumor growth will be monitored.

The Ethics Committee of Xinhua Hospital Affiliated to Shanghai Jiaotong University School of Medicine carried out a comprehensive assessment of the animals, including experimental purposes, the expected benefits and causing injury, death, etc.

The Ethics Committee concluded that this study was carried out in strict accordance with the recommendations in the Guide for the Care and Use of Laboratory Animals of the National Institutes of Health. The protocol was approved by the Committee on the Ethics of Animal Experiments of Xinhua Hospital Affiliated to Shanghai Jiaotong University (Permit Number: XHEC-E 2011-006). All surgery was performed under sodium pentobarbital anesthesia, and all efforts were made to minimize suffering.

RPMI-1640 cell culture medium, fetal bovine serum (FBS), and serum-free medium AIM-V were purchased from Invitrogen (Shanghai, China). Rabbit anti-human ABL and rat anti-human heat shock protein 70 (HSP70) antibodies were purchased from Santa Cruz Biotechnology (Shanghai, China). Recombinant mouse granulocyte-macrophage colony-stimulating factor (rmGM-CSF), recombinant human interleukin (rhIL)-4, and rhIL-2 were purchased from PeproTech (Shanghai, China). The CytoTox 96 non-radioactive cytotoxicity assay kit was purchased from Promega BioSciences (Shanghai, China). The K562 cell line was provided by the Shanghai Institute of Hematology. The L1210 cell line, an acute lymphoblastic leukemia cell line derived from DBA/2 mice, was purchased from the Shanghai Institute for Biological Science (Shanghai, China). Both cell lines were cultured in RPMI 1640 medium supplemented with 10% FBS. To prevent contamination with plasma EXOs, cells were transferred to serum-free medium (AIM V) and cultured for 24 h. This culture medium was then used as the source of EXOs. DBA/2 female mice were purchased from the Shanghai Laboratory Animal Center (Shanghai, China) and used at 6–14 weeks of age. Mice were allowed to adapt to their environment for 1 week before initiation of the experiments. During the course of the experiments, DBA/2 mice were maintained under standard environmental conditions with free access to food and water. DBA/2 mice were treated according to the guidelines of The Ethics Committee of Xinhua Hospital Affiliated to the Shanghai Jiaotong University School of Medicine.

### Generation and purification of EXOs *from leukemia cells*

Generation and purification of EXOs from leukemia cells were performed as previously described [19]. Briefly, the culture supernatants of K562 and L1210 cells were subjected to 4 successive centrifugations: 300×*g* for 5 min to remove whole cells, 1,200×*g* for 20 min, 10,000×*g* for 30 min to remove debris, and 100,000×*g* for 1 h to pellet EXOs. The LEX pellets were washed twice in a large volume of phosphate-buffered saline (PBS) and recovered by centrifugation at 100,000×*g* for 1 h. LEXs were purified using sucrose density gradient centrifugation [19]. Briefly, EXOs were underlain with 1.5 mL of a 30% sucrose/D_2_O density cushion (density 1.210 g/cm^3^) followed by ultracentrifugation at 100,000×*g* at 4°C for 1 h. Approximately 2 mL of the cushion was collected from the bottom of the tube and diluted in 50 mL of PBS. Finally, the EXOs were concentrated to a volume of 10 mL by centrifugation for 60 min at 1000×*g* in a pre-rinsed 100-kDa molecular weight cut-off Amicon Ultra capsule filter (Millipore, Billerica, MA, USA). The amount of recovered exosomal proteins was measured using the Bradford assay (Bio-Rad, Richmond, CA). EXOs of K562 and L1210 cells were termed LEX_K562_ and LEX_L1210_, respectively.

### Morphological characteristics of LEX_K562_

LEX_K562_ (10 μg) were washed in cacodylate buffer, fixed in 2.5% glutaraldehyde (Polysciences, Shanghai, China) in cacodylate buffer overnight at 4°C, dehydrated by graded alcohol processing, and flat embedded in LX-112 epoxy resin. Sections were cut with an ultramicrotome. Mounted sections were collected on copper grids, stained with a saturated solution of uranyl acetate, and submitted for observation and imaging under a Philips CM12 transmission electron microscope (TEM) [20].

### Detection of expression of ABL and HSP70 in LEX_K562_

The LEX_K562_ suspension (20 μL) was added to 20 μL of a 2% paraformaldehyde solution and incubated at room temperature for 1 h. Next, 3–6 μL of fixed EXOs was dripped onto a nickel grid, allowed to dry completely, and stained with diluted rat anti-human HSP70 and ABL antibodies. Samples were first incubated at room temperature for 30 min and then overnight at 4°C. Next, 25 μL diluted scintillation proximity assay (SPA) suspension was dripped onto a clean and flat hydrophobic membrane to form liquid drops. The grid was gently placed on the SPA drops with the film facing down, incubated at room temperature for 2 h, and then rinsed with PBS. Then, 5% uranyl acetate staining solution was dripped onto the nickel grid for negative staining and incubated at room temperature for 10 min. A blank control was included in which the primary antibody was replaced with PBS. EXO staining was visualized under TEM [19]. EXOs containing black colloidal gold particles on the extramembrane and cavum of the vesicles were considered to be positive.

To further confirm the expression of BCR-ABL and HSP70 in LEX_K562_, we performed western blotting as previously described [19]. Briefly, 10 μg of LEX_K562_ and K562 cell extracts was re-suspended in sodium dodecyl sulfate buffer and heated at 95°C for 5 min. Then, 0.13 M dithiothreitol was added to the samples, and they were subjected to 7.5% sodium dodecyl sulfate polyacrylamide gel electrophoresis. Following electrotransfer to nitrocellulose membranes, blocking was performed with 5% bovine serum albumin at room temperature for 2 h. Rabbit anti-human HSP70 and ABL antibodies were added separately, and the blots were incubated at room temperature for 1 h. Then, blots were incubated for 1 h with horseradish peroxidase-labeled secondary antibodies. Proteins were visualized by enhanced chemiluminescence substrate, and the blots were developed with the Pico West illumination kit (Promega, Shanghai, China).

### Generation of DCs

DBA/2 murine bone marrow-derived DCs were generated from bone marrow cells cultured in the presence of GM-CSF and IL-4 as previously described [17]. Briefly, bone marrow cells from the femurs and tibias of mice were flushed with RPMI 1640 medium. Red blood cells were depleted with 0.84% ammonium chloride, and the cells were plated in DC culture medium containing 10% FBS, GM-CSF (10 ng/mL), and IL-4 (10 ng/mL). On day 3, non-adherent cells, including granulocytes and T- and B-lymphocytes, were gently removed, and fresh medium containing GM-CSF and IL-4 was added. Two days later, loosely adherent proliferating DC aggregates were dislodged and replated. On day 7, non-adherent DCs were harvested and matured by incubation with 1 μg/mL lipopolysaccharide (Sigma-Aldrich, Shanghai, China) for 6 h. DCs were then harvested for further use.

### LEX uptake by DCs *in vitro*

Our previous study demonstrated that DC-derived EXOs are taken up by DCs *in vitro*. In the present study, we investigated whether LEXs are taken up by DCs *in vitro*. LEXs were stained with carboxyfluorescein succinimidyl ester (CFSE) and then co-cultured with DCs. CFSE-positive cells were visualized at different time points for up to 10 h by confocal microscopy. To investigate the rate of decay, DCs incubated with LEX_K562_ for 4 h were washed twice with PBS, further cultured in media, and then visualized by confocal microscopy at different time points for up to 72 h. DCs pulsed with LEX_K562_ and LEX_L1210_ were termed DC/LEX_K562_ and DC/LEX_L1210_, respectively.

### Animal study

To examine the ability of LEX_L1210_ and DC/LEX_L1210_ to induce protective antitumor immunity, DBA/2 mice were randomly divided into 4 groups (n = 8 per group). The animals were immunized subcutaneously (s.c.) on the inner side of their thighs with the following: PBS (control), LEX_L1210_ (30 μg), non-pulsed DCs (1×10^6^/mouse), and different doses of DC/LEX_L1210_ (1.0−4.0×10^6^ cells/mouse). On days 7–10 after immunization, all mice were challenged with L1210 leukemia cells on the outer side of the same thighs (0.5×10^6^ cells/mouse). To determine immune specificity, after immunization, a group of tumor-free mice were challenged s.c. with P388 cells, another DBA/2 mouse leukemia cell line (Shanghai Institute for Biological Science) (5×10^5^ cells/mouse). Tumor growth was monitored daily for up to 4 weeks using a caliper. For ethical treatment of the animals, all mice were euthanized when the tumor diameter reached 1.5 cm.

To examine the therapeutic effect on established tumors, DBA/2 mice (n = 8 per group) were s.c. inoculated with L1210 cells (0.5×10^6^ cells/mouse). After 5 d, when the tumors became palpable (∼5 mm in diameter), mice were s.c. immunized with LEX_L1210_ and different doses of DC/LEX_L1210_ (1.0−4.0×10^6^ cells/mouse). Animal mortality and tumor growth or regression were monitored daily for up to 10 weeks; for ethical treatment of the animals, the mice were euthanized when the tumor diameter reached 1.5 cm.

### Cytotoxicity assay

Cytotoxic responses were evaluated by lactate dehydrogenase (LDH) release using the CytoTox 96 cytotoxicity assay kit according to the manufacturer's instructions. Splenic T-cells from mice immunized intravenously with LEX_L1210_, DC/LEX_L1210_, and PBS (control) were harvested and purified over nylon wool. Cells were prepared at 1.5×10^6^/mL in complete medium. They were then co-cultured with 1.5×10^5^ irradiated L1210 cells in 100-mm Petri dishes containing 100 U/mL of rhIL-2 at 37°C for 6 d. On days 2 and 5, rhIL-2 was added. Cells were harvested at the end of the culture period, and viable T-cells were isolated using Ficoll-Paque centrifugation (Pharmacia Biotech, Shanghai, China). These cells were thereafter referred to as effector cells. The LDH assay is an enzymatic method that colorimetrically quantifies LDH released from lysed target cells. L1210 and P388 cells served as controls and were mixed at different ratios with effector cells after incubation for 4 h at 37°C. The spontaneous/maximal release ratio was <20% in all experiments. Specific lysis (%) was calculated as follows: (experimental LDH release – effector cell spontaneous LDH release – target spontaneous LDH release)/(target maximum LDH release)×100.

### Statistical analysis

For the mouse study, the Kaplan-Meier product-limit method was used to calculate survival rates. Differences between groups were determined using the generalized Log-rank test. Survival data are also presented as median survival time (MST), which is the time point at which half of the mice were alive. Data have been expressed as X±S; statistical analysis was performed using the Student's *t*-test. For all statistical analyses, *P*<0.05 was considered statistically significant.

## Results

### Leukemia cells secrete exosomes

**Figure 1:**
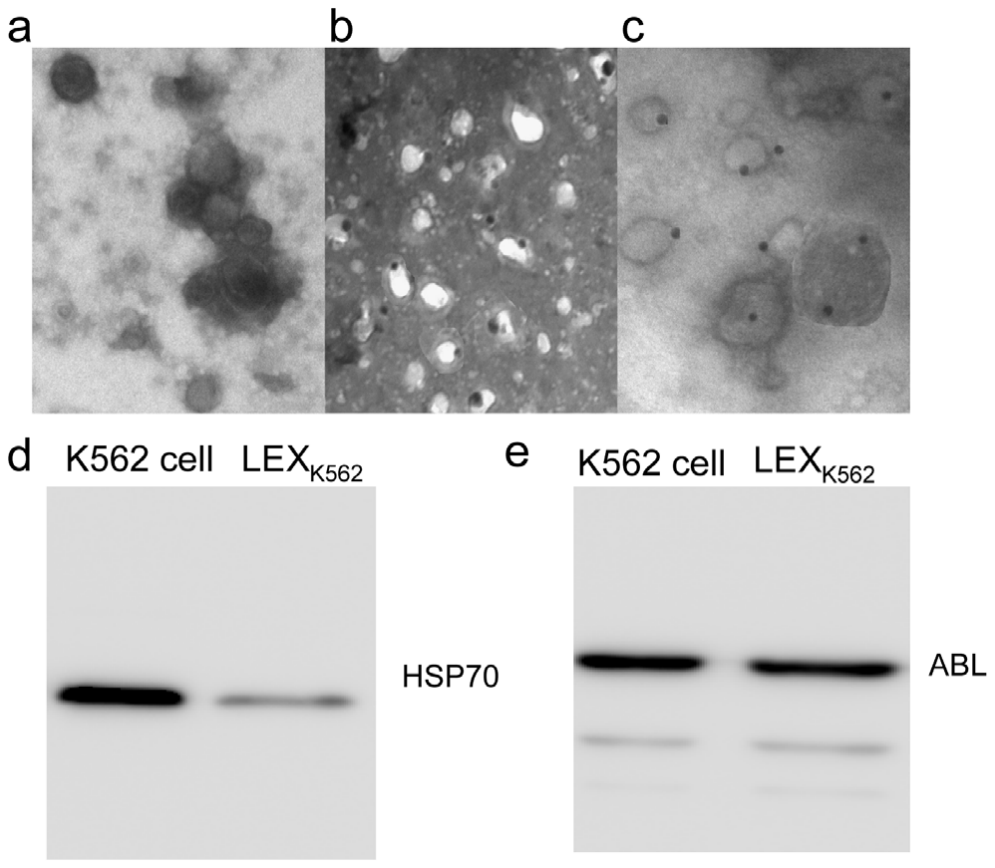
Morophology and expression of heat shock protein 70 and ABL in LEX_K562_. (a) Transmission electron micrograph of K562 cell-secreted exosomes (×100K). (b and c) Electron micrograph of heat shock protein 70 (HSP70)- and ABL-labeled exosomes. (d) Western blot analysis demonstrating the presence of HSP70 and ABL molecules in K562 cells and K562-derived exosomes (LEX_K562_).

We first assessed whether the K562 cell line releases EXOs similar to other tumor cell lines. When the K562 culture supernatant was differentially centrifuged up to 100,000×*g*, the pellet was similar to previously reported EXOs, as determined by electron microscopy. The vesicles of these preparations were <100 nm in diameter and had the dimpled, cup-shaped morphologies characteristic of EXOs (Fig. 1a). In order to explore whether LEX_K562_ harbored specific proteins from its parental K562 cells, we analyzed the expression of BCR-ABL, a fusion protein in K562 cells, using anti-ABL antibodies in LEX_K562_. More than 60% of LEX_K562_ were positively stained for ABL (Fig. 1c and 1e), indicating that LEXs harbor proteins of their parental leukemia cells. To further confirm this finding, we performed a western blot to analyze ABL expression in LEX_K562_. Our data suggested that LEX_K562_ harbored BCR-ABL molecules expressed in K562 cells (Fig. 1d and 1e). In addition, LEX_K562_ expressed HSP70 (Fig. 1b and 1d), a chaperone protein involved in the induction of immunity. we also examined ER-residing protein Grp94 in exosomes derived from K562 leukemia cells by western blot and our data showed that ER-residing protein Grp94 was absent in LEX_K562_ (Figure S1). Acetylcholinesterase activity, a characteristic enzyme in reticulocyte-derived exosomes also were detectable in LEX_K562_ (data not shown). Taken together, these results indicated that vesicles obtained from cell-free supernatants of K562 leukemia cells exhibited biophysical properties of exosomes. and LEXs contain known leukemia cell associated antigens as well as HSP70, a molecule that facilitates antigen presentation and CTL induction. Together, these data indicate that LEXs are a potential source of leukemia cell-associated antigens.

### LEXs are taken up by DCs

**Figure 2:**
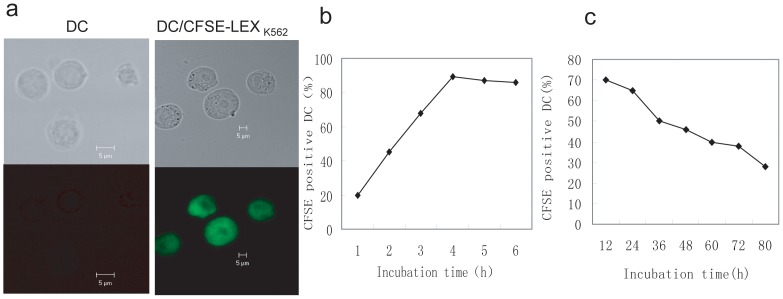
Exosome-uptaking by dendritic cells (a) Carboxyfluorescein succinimidyl ester (CFSE)-labeled exosomes were co-cultured *in vitro* with dendritic cells (DCs), and CFSE-positive DCs were detected using flow cytometry at different times during the culture. Confocal microscopy was used concurrently with flow cytometry to visualize the cultured DCs. (b) Phase changes of CFSE expression in DCs at different time points during the culture. (c) To investigate the rate of decay of exosomes (EXOs) in DCs, DCs were incubated with CFSE-labeled EXOs for 4 h, washed twice with phosphate-buffered saline, cultured in culture medium, and examined at different time points for up to 72 h.

To determine whether LEX_K562_ are taken up by DCs and to understand the kinetics of DCs sensitized by LEX_K562_ *in vitro*, LEX_K562_ were labeled with CFSE and then co-cultured with DCs. CFSE expression in DCs was tested at different time points using flow cytometry and confocal fluorescence microscopy. CFSE-positive DCs (23%) were detectable as early as 1 h after incubation (Figure 2a and 2b). The number of CFSE-positive cells (86.5%) reached a plateau 3–4 h after incubation. To investigate the rate of decay of LEX_K562_ in DCs, DCs were incubated with CFSE-labeled LEX_K562_ for 4 h, washed twice with PBS, further cultured in culture medium, and then examined at different time points for up to 72 h. The number of CFSE-positive DCs decreased over time (Fig. 2c). CFSE-positive DCs (18%) remained detectable at 72 h after culturing, indicating that the uptake of LEX_K562_ by DCs is stable.

### LEX-pulsed DCs induced a strong cytotoxic antileukemic immune response *ex vivo*

**Figure 3:**
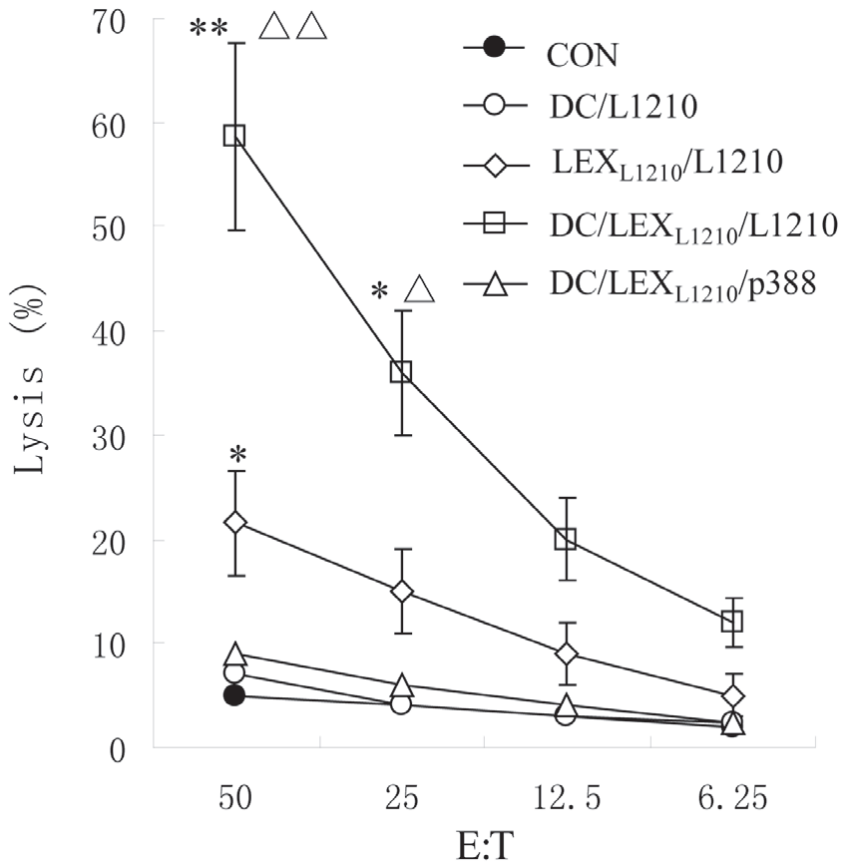
Cytotoxicity assay. Cytotoxic responses were evaluated by the lactate dehydrogenase (LDH)-releasing method. Splenic T-cells from mice immunized intravenously with L1210-derived exosomes (LEX_L1210_) and dendritic cells (DCs) pulsed with LEX_L1210_ or phosphate-buffered saline (PBS) as a control were harvested and co-cultured with irradiated L1210 cells. At the end of the culture period, viable T-cells were separated using Ficoll-Paque centrifugation and were thereafter referred to as effector cells. The LDH assay is an enzymatic method used to colorimetrically quantify LDH released from lysed target cells, including L1210 or P388 cells, which served as the control and were mixed at different ratios with effector cells after incubation for 4 h at 37°C. The spontaneous/maximal release ratio was <20% in all experiments. Specific lysis (%) was calculated as follows: (experimental LDH release – effector cell spontaneous LDH release – target spontaneous LDH release)/(target maximum LDH release)×100. * *P*<0.05 compared with the PBS, DC, and P388 groups; ** *P*<0.01 compared with the PBS control group; △ *P*<0.05 compared with the LEX_L1210_ group; △△ *P*<0.01 compared with the LEX_L1210_ group. Experiments were performed in triplicate. One representative experiment is shown.

In order to examine whether LEXs induced an antileukemic CTL immune response, L1210 leukemia cells were implanted in DBA/2 mice as an animal model of tumor growth. Splenic T-cells were isolated from mice immunized with PBS (control), non-pulsed DCs, LEX_L1210_, and DC/LEX_L1210_. T cells from mice immunized with PBS and non-pulsed DCs did not show killing activity against L1210 cells, whereas T cells from LEX_L1210_-immunized mice showed weak killing activity against L1210 cells (23.5%±3.21%; E:T ratio, 50:1) (Fig. 3). Interestingly, T-cells from DC/LEX_L1210_-immunized mice showed significantly stronger killing activity (57.15%±6.13%; E:T ratio, 50:1) than T-cells from LEX_L1210_-immunized mice (*P*<0.01). Thus, LEX-pulsed DCs induce a more potent antileukemic CTL response. Further, LEX_L1210_- and LEX_L1210_-pulsed DCs induce specific antileukemic effects against L1210 cells, as evidenced by the lack of killing activity in T-cells from DC/LEX_L1210_-immunized mice against P388 leukemia cells (Fig. 3).

### LEX-pulsed DCs induce strong protective immunity against leukemia cells

**Figure 4:**
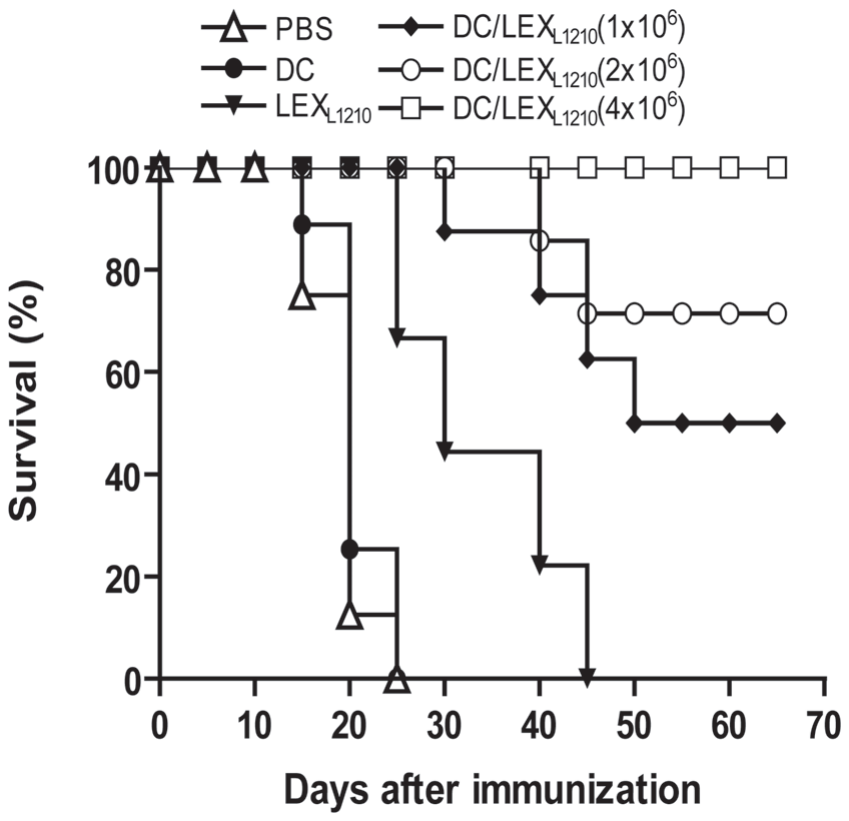
LEX_L1210_ and LEX_L1210_-pulsed DCs induce anti-leukemia protective immunity against L1210 leukemia cells. Survival of mice prophylactically immunized with different vaccines. DBA/2 mice (n = 8 per group) were immunized with phosphate-buffered saline (PBS), L1210-derived exosomes (LEX_L1210_), non-pulsed dendritic cells (DCs), and different doses of LEX_L1210_-pulsed DCs (DC/LEX_L1210_). On days 7–10 after immunization, all mice were challenged with L1210 leukemia cells. Vaccination with non-pulsed DCs (median survival time [MST], 20 d) did not significantly prolong the survival of mice as compared to the non-vaccinated PBS group (MST, 15 d), whereas LEX_L1210_ vaccination (MST, 30 d) improved survival as compared to the control group of non-vaccinated mice (*P*<0.05). In contrast, mice receiving different doses of DC/LEX_L1210_ (particularly 2×10^6^ and 4×10^6^) had significantly improved survival as compared to non-vaccinated mice (*P*<0.0001). Results were combined from 2 separate experiments.

Because the strength of *ex vivo* CTL cell responses was comparable between mice immunized with LEX_L1210_ and DC/LEX_L1210_, we examined the immune protection conferred by these 2 vaccines *in vivo*. To do so, we evaluated the efficacy of vaccination with LEX_L1210_ and DC/LEX_L1210_ in preventing tumor growth. L1210 leukemia cells were implanted in DBA/2 mice as an animal model of tumor growth. DBA/2 mice were immunized s.c. with PBS (control), LEX_L1210_, non-pulsed DCs, and different doses of DC/LEX_L1210_. On days 7–10 after immunization, all mice were challenged with L1210 leukemia cells. All mice injected with PBS and non-pulsed DCs showed tumor growth (100%), while only half (50%) of mice injected with LEX_L1210_ showed tumor growth (Table 1), indicating that LEXs induce protective immunity against leukemia cells. Interestingly, 87.5% (7/8) of DC/LEX_L1210_-immunized mice were tumor-free, indicating that LEX-pulsed DCs induce stronger antileukemic immunity than LEX alone. In addition, our data demonstrated that LEX_L1210_ and DC/LEX_L1210_ induce specific antileukemic immunity, because no protective immunity was observed in mice challenged with P388 leukemia cells. Moreover, our data also showed that immunization with LEX_L1210_ and DC/LEX_L1210_ significantly improved survival (Fig. 4). Vaccination with non-pulsed DCs (MST, 20 d) did not significantly prolong survival as compared to the non-vaccinated group (MST, 15 d), whereas LEX_L1210_ vaccination (MST, 30 d) improved survival as compared to the non-vaccinated group (*P*<0.05). In contrast, mice receiving different doses of DC/LEX_L1210_ (particularly 2×10^6^ and 4×10^6^) had a significantly improved survival as compared to non-vaccinated mice and LEX_L1210_-vaccinated mice, (*P*<0.0001). The MST was >60 d in 50%, 70%, and 100% of mice vaccinated with 1, 2, and 4×10^6^ DC/LEX_L1210_, respectively.

**Table 1:** Vaccination with LEXO and LEXO-targeted DC protects against tumor growth.

Vaccines	Tumor cell challenge	Incidence of tumor growth (%)
PBS	**L1210**	100% (8/8)
DC	**L1210**	100% (8/8)
LEX_L1210_	**L1210**	50% (4/8)
DC/LEX_L1210_	**L1210**	12.5% (1/8)
LEX_L1210_	**P388**	100% (8/8)
DC/LEX_L1210_	**P388**	100% (8/8)

To examine the antitumor immunity conferred by LEX_L1210_ and DC/LEX_L1210_, DBA/2 mice were randomly divided into 4 groups (n = 8) and immunized s.c. with the following vaccines on the inner side of their thighs: PBS (control), LEX_L1210_ (30 μg), unpulsed DCs (1×10^6^ cells), and LEX_L1210_-pulsed DCs (DC/LEX_L1210_) (1×10^6^ cells). On day 7 after immunization, all mice were challenged with L1210 leukemia cells on the outer side of the same thighs (0.5×10^6^ cells/mice). To determine immune specificity, a group of tumor-free mice after immunization, were challenged s.c. with p388 cells (5×10^5^ cells/mouse). Tumor growth was monitored daily for up to 4 weeks using a caliper. For ethical treatment of the animals, all mice were euthanized when the tumor diameter reached 1.5 cm. Three total experiments were performed. One representative experiment is shown.

**Figure 5:**
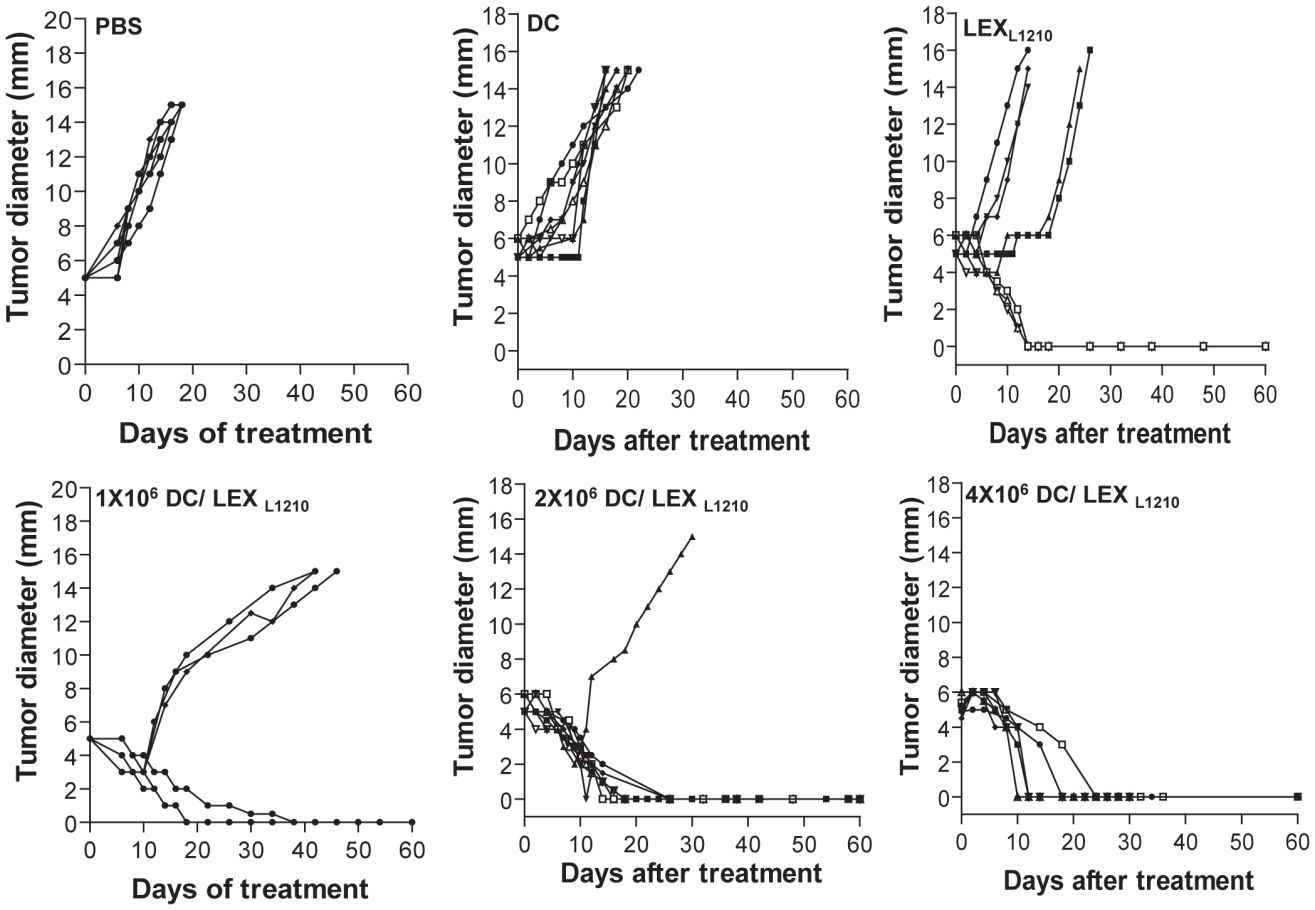
Therapeutic effect of LEX_L1210_-pulsed DCs on established tumors. To examine the therapeutic effect on established tumors, DBA/2 mice (n = 8 per group) were subcutaneously (s.c.) inoculated with L1210 cells (0.5×10^6^ cells/mouse). After 5 d, when tumors became palpable (∼5 mm in diameter), mice were s.c. immunized with L1210-derived exosomes (LEX_L1210_) and different doses of dendritic cells (DCs) pulsed with LEX_L1210_ (DC/LEX_L1210_) (1.0–4.0×10^6^ cells/mouse). Animal mortality and tumor growth or regression were monitored daily for up to 10 weeks. For ethical treatment of the animals, mice were euthanized when the tumor diameter reached 1.5 cm. Experiments were performed in triplicate. One representative experiment is shown.

To further examine the therapeutic effect of LEX_L1210_ and DC/LEX_L1210_, mice bearing palpable tumors (∼5 mm in diameter) were immunized with LEX_L1210_ and DC/LEX_L1210_. All mice in the control group died at approximately 18 d after immunization, and 5 of 8 mice in the LEX_L1210_ group died (Fig. 5). However, immunization with DC/LEX_L1210_ dose-dependently protected mice from established-tumor growth, because tumor retarded were observed in 5/8 (62.5%), 7/8 (87.5%), and 8/8 (100%) of tumor-bearing mice, which immunized with 1×10^6^, 2×10^6^, and 4×10^6^ DC/LEX_L1210_, respectively. Taken together, these data indicate that LEX_L1210_-pulsed DCs more efficiently induce protective antileukemic immunity than LEX_L1210_ alone.

## Discussion

Recent studies have demonstrated that TEXs harbor tumor cell-associated antigens and can induce antitumor immunological effects [7], [11]. Wolfers *et al*. [12] reported that the morphological characteristics and density of TEXs are similar to those of DEXs. TEXs can be isolated and purified from the supernatant of tumor cell cultures, blood, and malignant effusions [11], [12], [21]. TEXs also harbor major histocompatibility complex class I antigens (MHC-I), lysosome-associated membrane glycoprotein 1, HSP70, and other tumor-related antigens. They can induce immunological responses and antitumor immunological effects of T-cells that are restricted by tumor antigen-specific MHC-I. Therefore, TEXs may be a source of tumor antigens for tumor immunotherapy.

Leukemia cells, which originate from hematopoietic cells, also secrete EXOs. Although K562 chronic myelogenous leukemia cells [22], [23] have been reported to produce EXOs, little is known about their role in the biology of chronic myelogenous leukemia. In this study, EXO_K562_ were systemically characterized by electron microscopy, confocal microscopy, and flow cytometry. We demonstrated that EXO_K562_ expressed membrane molecules derived from K562 cells but at significantly lower levels than expressed by K562 cells (data not shown). TEM analysis and western blotting indicated that EXO_K562_ expressed HSP70 and ABL proteins. Two-dimensional protein electrophoresis revealed that EXO_K562_ harbor most proteins expressed in K562 cells although at lower levels. Interestingly, some proteins were expressed at higher levels in EXO_K562_ than in K562 cells (unpublished data), suggesting that EXO_K562_ preparation enriches for some proteins from the parental K562 cells. The specific proteins that are enriched remain to be determined using proteomic techniques, such as mass spectrometry.

Membrane transfer occurs in systems that do or do not require cell-to-cell contacts [24]. Knight *et al*. reported that DCs acquire antigens from cell-free DC supernatants [25]. In the present study, we demonstrated that EXOs are taken up by DCs.

Among antigen-presenting cells, DCs most potently initiate cellular immune responses through stimulating naive T-cells. The action of DCs mainly results from constitutive upregulated expression of adhesion molecules, MHC, and costimulatory molecules [26]. DCs play a central role in various immunotherapies by generating CTL. Accordingly, DC-based vaccines have been successfully used for cancer prophylaxis [27] and some murine tumor models [14], [16], which has provided a basis for using DCs in human anticancer vaccinations. Several strategies have been investigated for DC loading with tumor cells [28], [29], including transfecting DCs with RNA encoding TAA [30], [31], acid-eluted tumor peptides [32], [33], and TEXs [16], [17], [34], [35].

EXO_K562_ and K562 cell lysates induced CTL activity, but relatively weakly. Previous studies indicated that TEXs might depress the host's immunologic response [15], which may limit immunotherapy using TEX-based vaccines. Therefore, it is important to overcome these drawbacks of TEXs and enhance their antitumor effects in order to develop highly effective TEX-based tumor vaccines. In our previous study, we demonstrated that EXO-pulsed DCs induce stronger antitumor immunity than EXOs and DCs alone [17]. In the present study, we demonstrated that EXO_K562_-pulsed DCs activate CTLs *in vitro*, which kill target cells more potently than CTLs induced by EXO_K562_ alone or by DCs pulsed with cell lysates. To further confirm whether EXO_K562_ and EXO_K562_-pulsed DCs have an effect *in vivo*, we performed preliminary experiments in an animal model. Our data demonstrated that LEXs induce antileukemic immunity and that LEX-pulsed DCs had more potent antigen-specific antileukemic effects, because all mice injected with non-pulsed DCs developed tumors. Therefore, we conclude that LEXs are an important source of leukemia cell antigens and that LEX-pulsed DCs represent a new, highly effective DC-based vaccine for the induction of antileukemic immunity.

## Supporting Information

Figure S1 **Detection of expression of Grp94 in K562-derived exosomes.** Western blot analysis demonstrating the presence of ER-residing protein Grp94 in K562 cells and K562-derived exosomes (LEX_K562_).
(PDF)
