# Supplementary figures and images for: Dendritic Cells Pulsed with Leukemia Cell-Derived Exosomes More Efficiently Induce Antileukemic Immunities
Source: PLoS One. 2014 Mar 12;9(3):e91463. doi: 10.1371/journal.pone.0091463 (PMC3951359; doi:10.1371/journal.pone.0091463)

**Figure S1. Ye Yao, et al.**

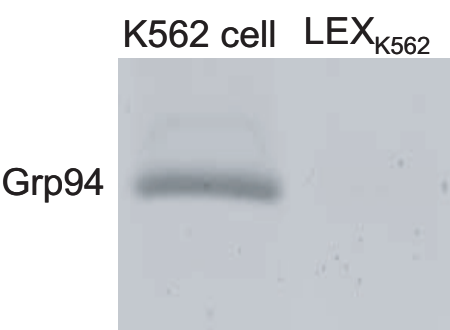

Supplement: Figure S1 — Detection of expression of Grp94 in K562-derived exosomes. Western blot analysis demonstrating the presence of ER-residing protein Grp94 in K562 cells and K562-derived exosomes (LEXK562). (PDF) [file pone.0091463.s001.pdf]
